# Intravital and Whole-Organ Imaging Reveals Capture of Melanoma-Derived Antigen by Lymph Node Subcapsular Macrophages Leading to Widespread Deposition on Follicular Dendritic Cells

**DOI:** 10.3389/fimmu.2015.00114

**Published:** 2015-03-13

**Authors:** Federica Moalli, Steven T. Proulx, Reto Schwendener, Michael Detmar, Christoph Schlapbach, Jens V. Stein

**Affiliations:** ^1^Theodor Kocher Institute, University of Bern, Bern, Switzerland; ^2^Institute of Pharmaceutical Sciences, Swiss Federal Institute of Technology, ETH Zürich, Zürich, Switzerland; ^3^Institute of Molecular Cancer Research, University Zürich, Zürich, Switzerland; ^4^Department of Dermatology, Inselspital – Universitätsspital Bern, Bern, Switzerland

**Keywords:** tumor-draining lymph node, tumor-derived antigen deposition, follicular dendritic cells, intravital two-photon imaging, whole-organ imaging

## Abstract

Aberrant antigens expressed by tumor cells, such as in melanoma, are often associated with humoral immune responses, which may in turn influence tumor progression. Despite recent data showing the central role of adaptive immune responses on cancer spread or control, it remains poorly understood where and how tumor-derived antigen (TDA) induces a humoral immune response in tumor-bearing hosts. Based on our observation of TDA accumulation in B cell areas of lymph nodes (LNs) from melanoma patients, we developed a pre-metastatic B16.F10 melanoma model expressing a fluorescent fusion protein, tandem dimer tomato, as a surrogate TDA. Using intravital two-photon microscopy (2PM) and whole-mount 3D LN imaging of tumor-draining LNs in immunocompetent mice, we report an unexpectedly widespread accumulation of TDA on follicular dendritic cells (FDCs), which were dynamically scanned by circulating B cells. Furthermore, 2PM imaging identified macrophages located in the subcapsular sinus of tumor-draining LNs to capture subcellular TDA-containing particles arriving in afferent lymph. As a consequence, depletion of macrophages or genetic ablation of B cells and FDCs resulted in dramatically reduced TDA capture in tumor-draining LNs. In sum, we identified a major pathway for the induction of humoral responses in a melanoma model, which may be exploitable to manipulate anti-TDA antibody production during cancer immunotherapy.

## Introduction

Melanoma, a potentially lethal skin cancer, is often associated with the induction of humoral responses, which are typically inversely correlated with the extent of the disease (1, 2). While some controversy exists on whether anti-tumor-derived antigen (TDA) Ig responses enhance or hamper tumor growth and metastasis (1–4), there is renewed interest in investigating how interactions between the adaptive immune system and cancer cells shape the spread versus control of primary tumors (5–7). There are two major factors underlying this surge of interest: first, comprehensive patient studies have shown that the presence of adaptive immune cells in tumors positively correlates with a beneficial prognosis (8, 9). As example, tumor infiltration of follicular helper T cells, which are required for high affinity Ig production by germinal center (GC) B cells, are associated with a good prognosis (10, 11). Similarly, CD8^+^ T cell infiltration into breast tumors is associated with survival when analyzed in more than 12,000 patients (12). Second, preclinical and clinical studies have identified a promising antitumoral efficacy by blocking immunosuppressive molecules such as PD-1 and CTLA-4, which overrides inhibitory signals and boosts host cytotoxic T cell responses against cancer cells (7, 13). Importantly, a recent study has shown that antibodies directed against both surface and cytoplasmic molecules contribute to efficient CD8^+^ T cell responses by forming immune complexes that are efficiently taken up by dendritic cells (DCs) for presentation to cytotoxic T cells (14). In addition, antibodies directed against intracellular tumor antigens are able to directly kill cancer cells (15). Thus, anti-TDA Ig may exert multiple effects on tumor control, beyond direct killing or antibody-dependent cytotoxicity.

Yet, there is surprisingly little information on how TDA induces humoral responses. For example, it remains unclear whether serum anti-TDA Ig levels observed in mouse cancer models and in patients are exclusively initiated or maintained in tumor-draining lymph nodes (TDLNs) or whether metastatic spread precipitates such systemic responses. Furthermore, how TDA is actually collected by TDLNs is not well defined. Studies addressing antimicrobial responses have analyzed the uptake of unprocessed Ag, using as tools fluorescently labeled immune complexes, virus particles, or other fluorescent cell populations, often in combination with intravital two-photon microscopy (2PM) (16). These findings have identified at least three pathways of Ag transport to lymphoid tissue. First, B cells have been shown to become directly activated by DCs that have migrated from the periphery into draining lymphoid tissue and carry unprocessed Ag on their surface (17). Second, low-sized lymph-borne Ag is directly delivered to follicular dendritic cells (FDCs) via a conduit system formed by stromal cells of the lymph node (LN) interstitium (18). FDCs are arguably the most important antigen-presenting cells for naïve B cells and play a central role for the establishment and maintenance of GC reactions, which enhance the affinity of mAbs through somatic hypermutation and selection of suitable clones (19). Third, lymph-borne antigens including virus particles are captured by macrophages located in the subcapsular sinus (SCS), which are equipped with scavenging receptors such as lectins (20–22). In this context, it is interesting to note that CD169^+^ SCS macrophages also mediate the uptake of TDA from apoptotic tumor cells, which they then process for presentation on peptide–MHC complexes. This leads to T cell activation and subsequent tumor elimination (23). In addition, SIGN-R1^+^ DC subsets located in the LN medulla participate in the capture of specific virus particles arriving in afferent lymph (24). These cells then relay captured antigen to B cell follicles, and when present as immune complexes, non-specific B cells act as transporters for final deposition on FDCs (25).

Yet, which of these multiple pathways is engaged in TDA capture and relay to FDCs for B cell activation remains little investigated. This is in large part owing to a lack of precise detection and quantification methods of unprocessed TDA in commonly used immunohistological analysis. Based on the successful adoption of intravital 2PM in observing and quantifying the uptake of fluorescent viral particles and immune complexes in reactive LNs (16), we set out to perform similar experiments in TDLNs, using a B16.F10 melanoma model expressing the fusion protein tandem dimer tomato (tdTom) as a surrogate TDA for use in fluorescent imaging (26, 27). We combined dynamic time-lapse 2PM imaging with stereomicroscopy and quantitative optical projection tomography (OPT) of whole-mount TDLNs. OPT is based on reconstruction of back-projected images to form 3D images and has proven a useful tool for isotropic imaging and quantification of B cell follicles and vascular networks in LNs (28). We found that systemic anti-TDA IgG responses correlated with an unexpectedly widespread distribution of TDA in the vast majority of FDC networks of TDLNs, and also in part in further draining LNs but not in non-draining LNs. Furthermore, we show that SCS macrophages are required for the rapid uptake of subcellular-sized tdTom^+^ TDA, from where it is transported to FDCs for extensive screening by B cells. In sum, we propose a mechanism of how TDA is processed in TDLNs to induce local B cell responses that lead to systemic IgG levels. Furthermore, this is to the best of our knowledge the first study providing a quantitative assessment of Ag deposition in whole-mount lymphoid tissue using 3D reconstructions.

## Materials and Methods

### Mice

Four to 6-week-old female C57BL/6 mice were purchased from Janvier (AD Horst, Netherlands). Igh-J^tm1Cgn^ B cell-deficient (JHT) mice (29) and C57BL/6-Tg(CAG-EGFP)1Osb/J ubiquitously GFP-expressing mice were obtained from the Institut für Labortierkunde, University of Zürich, Switzerland. Tg(IghelMD4)4Ccg BCR-transgenic (30) mice on a RAG^−/−^ background (MD4) were kindly provided by Emilie Jacque, NIMR, London, UK. CX3CR1^tm1Litt^ fractalkine receptor-heterozygous (CX3CR1^+/gfp^) mice (31) were kindly provided by Prof. Richard Ransohoff (Lerner Research Institute, Cleveland, OH, USA). All mice were kept under SPF conditions at the Central Animal facility at the Department for Clinical Research and the Theodor Kocher Institute, University of Bern. All animal work has been approved by the Cantonal Committee for Animal Experimentation and conducted according to federal and cantonal guidelines.

### Antibodies and reagents

APC-conjugated anti-Ly6C (HK1.4) and PerCP-conjugated anti CD11b (M1/70) were from Biolegend. Purified anti-mouse-LYVE-1 was purchased from R&D. Purified anti-CD35 mAbs (7G6) was purchased from BD Biosciences. Anti-PNAd mAb (MECA-79) was affinity purified from hybridoma supernatant (Nanotools). All mAbs were fluorescently conjugated using AlexaFluor protein labeling kits (Molecular Probes, Invitrogen). CellTracker Blue (CMAC) fluorescent dye for cell labeling was also purchased from Molecular Probes.

### Tumor cell line and induction of s.c. tumors

We have previously described the stable murine melanoma cell line B16.F10-tdTom expressing a fusion protein of SNAP-tag and tdTomato red fluorescent protein and coexpressing luciferase (27). Single B16.F10-tdTom stable clones were isolated by limiting dilution and tdTomato expression was analyzed by flow cytometry (FACScalibur, BD Bioscience). Aliquots were frozen and stored in liquid nitrogen to avoid maintaining the cell line in culture with high passage numbers. Before each tumor injection experiment, a fresh aliquot of cells was thawed and cultured in Dulbecco’s modified Eagle’s medium (DMEM) (Invitrogen) containing 10% fetal calf serum (FCS) and 1.2 mg/ml G418 (Invitrogen). tdTomato fluorescent intensity was verified each time by flow cytometry before *in vivo* injection. Adult mice (6–8 weeks) received s.c. injections of 2 × 10^5^ B16.F10-tdTom cells in 10 μl to trigger tumor formation in the right footpad. Tumor growth was observed over a period of up to 30 days and where indicated observed under a stereomicroscope setup equipped with a fluorescent light source and a CCD camera (Leica MZ16; filters: GFP excitation 480/40 nm, emission 510; Cy3 excitation 560/40 nm, emission 605). In some experiments, we injected 20 μl of B16.F10-tdTom tumor lysate (10^7^ cells sonicated in 1 ml of 15 mM carbonate buffer, pH 9.6, at constant cycles for 15 s) in the right footpad. For macrophage depletion, mice were treated with control liposomes or CLL (2 mg in 200 μl PBS) i.p. 24 h after tumor cell injection in the footpad, and then treated every 2 days with 1 mg of control liposomes or CLL i.p. for 15 days.

### ELISA for detection of anti-TDA IgG

Serum of control mice and mice bearing B16.F10-tdTom tumors were collected for IgG titration. In brief, NuncTM 96-Well plates were plated with 100 μl of B16.F10-tdTom lysate diluted 1:25–1:78125 o.n. at 4°C. After washing with washing buffer (WB; PBS/0.05% Tween20) (Sigma-Aldrich), plates were incubated with 300 μl WB/5% dry milk for 2 h at RT. Serum dilutions (100 μl/well in 1:5 steps) were added and incubated for 2 h at 37°C. After washing with WB, biotinylated polyclonal goat anti-mouse IgG (10 ng/well; AbD Serotec) diluted in WB was added for 1 h at RT. Wells were washed and incubated for 1 h at RT with 100 μl streptavidin-HRP (AbD Serotec) diluted 1/1000 in WB. Bound antibodies were detected using OPD substrate, and absorbance was read at a wavelength of 490 nm. Results were calculated as titer by interpolation of absorbance values at a fixed serum dilution into a linear regression analysis plotting.

### 2PM of popliteal PLNs

Where indicated, B cells were isolated from C57BL/6 mice using magnetic bead sorting (EasyStep negative isolation Kit, STEMCELL Technologies; purity of >95%) and labeled with CMAC as described (32). Labeled B cells (5 × 10^6^ cells/mouse) were injected i.v. into sex-matched tumor-bearing recipient mice 24 h before 2PM imaging. Mice were surgically prepared as described (33). In brief, mice were anesthetized by i.p. injection of physiologic saline solution containing xylazine (10 mg/kg) and ketamine (50 mg/kg). After shaving and fixation of the right hind leg, the tumor-draining popliteal LN was carefully exteriorized without directly touching it, and kept moist with saline. 2PM imaging was performed using the TrimScope system equipped with a 20× objective (NA 0.95) (LaVision Biotec, Germany) and a Ti:Sapphire NIR laser (MaiTai, Spectraphysics) tuned to 780 or 840 nm. For four-dimensional analysis of cell migration, 10–18 *z*-stacks (spacing 2–4 μm) of 200–300 μm × 200–300 μm *x*–*y* sections were acquired every 20 s for 20 min. HEVs and blood vessels were identified by 10–15 μg AlexaFluor633-conjugated MECA-79 or Dextran Cascade blue (10 kDa, Molecular probes; 50 μl of 20 mg/ml stock solution per mouse) injected i.v. prior to 2PM observations. Alexa488-conjugated anti-LYVE-1 (0.1 mg/ml; Clone 223322, R&D systems) was s.c. injected into footpads in a volume of 20 μl 12 h prior to imaging. Sequences of image stacks were transformed into volume-rendered four-dimensional movies using Volocity or Imaris software, which was also used for semiautomated tracking of cell motility in three dimensions. For instantaneous 3D velocity and turning angle calculations, we used MatLab scripts as described (34).

### Optical projection tomography of control and tumor-draining LNs

Where indicated, GFP-expressing B cells from LNs and spleen were purified by negative immunomagnetic cell sorting and transferred i.v. into tumor-bearing recipient mice 1 day before PLN excision (2 × 10^7^/mouse). Excised LNs were carefully cleaned of surrounding fat, fixed in 4% ultrapure paraformaldehyde (Electron Microscopy Sciences, Hatfield, PA, USA) for 4 h at 4°C and then washed in PBS. LNs were embedded in 2% ultrapure agarose (Invitrogen) and incubated for 2 weeks in ScaleU2 solution (4 M urea, 30% glycerol, 0.1% Triton-X, pH 7.7), which preserves fluorescent protein signals (35). OPT scanning was performed according to the manufacturer’s instructions (Bioptonics). Filter sets were exciter 425/40, emitter LP475 for autofluorescent signal, exciter 480/20, emitter LP515 for green fluorescent signal and exciter 545/30, emitter 617/75 for red fluorescent signal. Raw data were converted into 3D voxel datasets using NRecon software from Bioptonics. Reconstructed virtual *xyz* data sets were exported as .TIFF or .bmp files and analyzed with IMARIS (Bitplane) for isosurface calculation of total LN volume and other parameters as described (28). IMARIS reconstructions were carefully adjusted to fit original NRecon reconstructions.

### Immunofluorescence of mouse PLNs

Contralateral and tumor-draining popliteal and inguinal LNs were removed, embedded in Tissue-tek (OCT compound, Syxmex Digitana) and snap-frozen in an isopentane bath (2-Methylbutane, Grogg Chemie) at −80°C. Cryostat sections (10 μm) were air-dried overnight before staining. Sections were fixed for 15 min with 1% paraformaldehyde at RT. After blocking with blocking buffer [5% skimmed milk (w/v), 0.3% Triton X-100 (v/v), and 0.04% (w/v) NaN_3_ in TBS], sections were incubated for 1 h at room temperature with the primary antibody diluted in PBS/1% FCS. Secondary antibodies were diluted in PBS/1% FCS and sections incubated for 1 h at room temperature before mounting in Mowiol (Calbiochem). Samples were analyzed using a Nikon Eclipse E600 microscope (Nikon Instruments) equipped with a Nikon Digital Camera (DXM1200F or DS-Ri1; Nikon Instruments). Images were processed with NIS Elements Version 3.2 software.

### Immunofluorescence of human lymph node samples

The study was approved by the Medical Ethics Committee of the Canton of Bern, Switzerland. Formalin-fixed LN tissue samples were obtained from patients with primary cutaneous melanoma (Brelsow index >1 mm) who underwent diagnostic sentinel LN biopsy. About 3 μm-thick paraffin-embedded sections were dewaxed, rehydrated, and pretreated as described previously (36). Tissue sections were serially incubated with a rabbit anti-human Melan A antibody (Novus, 110-57185) followed by incubation with a AlexaFluor594-labeled goat anti-rabbit F(ab′)2 (Invitrogen, A11072). Sections were then incubated with mouse-anti-human CD20 (Biosystem, PA0906) specific for naïve, memory, and GC B cells, followed by isotype-specific AlexaFluor488 goat anti-mouse IgG1 (Invitrogen, A-21121). Finally, cells were stained with diamidino-phenylindol nucleic acid stain (D1306; Invitrogen, Carlsbad, CA).

### Statistical methods

Data were analyzed with GraphPad Prism 6 using a Kruskal–Wallis test with Dunn’s multiple comparison test. Significance was set at *p* < 0.05.

## Results

### Immunofluorescent analysis of MelA co-localization with B cells in human TDLN sections

To establish whether TDA deposition is observed in human lymphoid tissue, we analyzed paraffin TDLN sections of human melanoma patients by immunofluorescent staining, focusing on MelA as model melanoma antigen. To identify B cell follicles, we labeled B cells directly using anti-CD20 mAb. While MelA staining readily identified metastasizing tumor cells (Figure S1 in Supplementary Material), we found that this antigen was variably co-localizing with B cells. Thus, while in some cases no co-localization of anti-CD20 and MelA signal was observed (Figure 1A in Supplementary Material), we found in other cases the presence of non-tumor cell associated MelA in B cell follicles (Figures S1B,C in Supplementary Material). Thus, TDA deposition is found in human TDLNs, where it is likely to contribute to the anti-IgG response observed in melanoma patients.

### Establishing a local tumor model leading to anti-TDA IgG responses in immunocompetent mice

**Figure 1:**
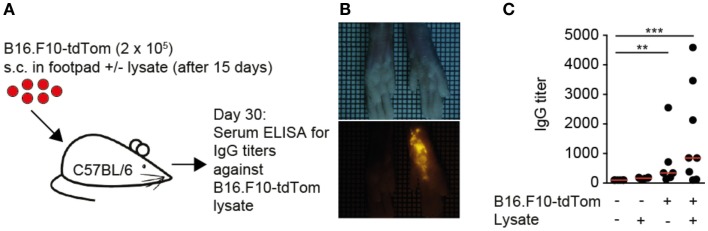
**Local melanoma tumors generate systemic circulating IgG levels**. **(A)**. Outline of local tumor injection experiment. B16.F10-tdTom^+^ cells were s.c. injected into C57BL/6 mice on day 0 and allowed to proliferate for 30 days. In some mice, B16.F10-tdTom lysate was additionally s.c. injected to test whether IgG production can be boosted. **(B)** Brightfield (top) and red fluorescent (bottom) stereomicroscopic images of tumor-injected and contralateral footpads on day 30. **(C)** IgG titers in control and tumor-bearing mice on day 30, with and without additional B16.F10-tdTom^+^ cell lysate injection for 15 days. Each dot represents the serum of one mouse. Data in **(C)** are pooled from two independent experiments and were analyzed by a Kruskal–Wallis test with Dunn’s multiple comparison test. **p < 0.01, ***p < 0.001.

Based on the observations made in human TDLNs, we set out to identify the mechanisms of TDA deposition using as melanoma model B16.F10 cells expressing high levels of a cytoplasmic tandem dimer Tomato fusion protein (B16.F10-tdTom) (27) (Figure 1A). Thirty days post s.c. injection into the footpad of immunocompetent C57BL/6 mice, we observed a bright red fluorescent signal in the footpad that had received B16.F10-tdTom cells, but not the contralateral footpad (Figure 1B). At this time point, we failed to detect fluorescent signals in lung, liver, kidney, and spleen, indicating that the primary tumor had not yet spread to other organs (not shown). Next, we investigated whether local tumor growth was sufficient to induce the generation of anti-tumor IgG production. We established an ELISA against B16.F10-tdTom melanoma cell lysate using an anti-tdTom^+^ mAb as positive control (not shown) before testing control and B16.F10-tdTom-tumor-bearing mice for the presence of anti-TDA mAbs in serum. At 30 days post B16.F10-tdTom injection, we readily detected significant titers of anti-B16.F10-tdTom IgG mAbs in tumor-bearing but not mock-injected mice (Figure 1C). In contrast, B16.F10-tdTom lysate injection 15 days post tumor implantation had only a minor, non-significant effect on anti-TDA IgG responses. In sum, we established a pre-metastatic local tumor model, which leads to robust anti-TDA IgG responses.

### Live imaging of tdTom^+^ TDA deposition on FDCs in TDLNs

**Figure 2:**
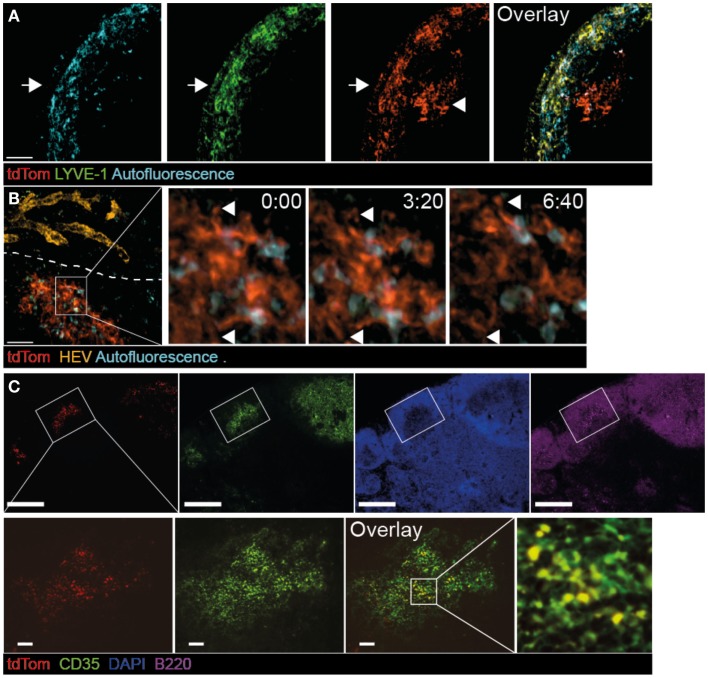
**Co-localization of tdTom^+^ signal with FDCs in TDLNs**. B16.F10-tdTom^+^ cells were s.c. injected into C57BL/6 mice on day 0 and allowed to proliferate for 30 days. Anti-LYVE-1 mAb was injected s.c. one day before 2PM analysis, while anti-PNAd (MECA-79) mAb was injected i.v. on the day of the 2PM experiment. **(A)** 2PM projection showing intranodal localization of tdTom^+^ signals (arrowhead) below the SCS region after LYVE-1 labeling. The arrow shows the outer capsule. Scale bar, 50 μm. **(B)** 2PM projection showing close proximity of tdTom^+^ structures with macrophages identified by autofluorescent signal and in regions that do not contain HEVs of the T cell area. The time-lapse sequence shows the dendrite-like shape of tdTom^+^ structures (arrowheads). Scale bar, 50 μm, time in minutes and seconds. **(C)** Immunofluorescent PLN section showing overlap of tdTom^+^ signals with the FDC-marker CD35 inside B cell follicles identified by B220 staining. Scale bars, 100 μm (top) and 20 μm (bottom).

The strong IgG response in metastatic-free tumor-bearing mice prompted us to investigate in detail the accumulation of TDA in TDLNs. To this end, we examined the deposition of fluorescent tdTom^+^ structures as surrogate TDA 30 days post tumor injection, using 2PM and immunofluorescence of TDLN sections. In some experiments, we co-transferred fluorescently labeled B cells, and stained lymphatic and blood vasculature with appropriate fluorescent markers (Figure S2A in Supplementary Material). 3D reconstructions of 2PM image sequences of TDLNs performed in live, anesthetized mice showed that in the SCS, tdTom^+^ structures were found closely associated with LYVE-1^+^ structures, which represent both lymphatic endothelial cells and macrophage subsets (Figure 2A, arrow). Strikingly, we also found tdTom^+^ signals below the SCS not co-localizing with LYVE-1 (Figure 2A, arrowhead and Movie S1 in Supplementary Material). Using anti-PNAd mAbs and fluorescent dextran as markers for HEVs and blood vessels, respectively, we found that tdTom^+^ structures were exclusively found in regions that did not contain HEVs (Figure 2B) but were transversed by extensive capillary networks (not shown). High-resolution 2PM image sequences uncovered that tdTom^+^ structures showed a dendritic morphology but did not undergo cell displacement, despite occasional shape changes of their dendrites (Figure 2B; Movies S1 and S2 in Supplementary Material). Based on their morphology and localization within TDLNs, we hypothesized that tdTom^+^ TDA is deposited on or endocytosed by FDC (37). This was indeed the case, since we found an almost complete overlap of tdTom^+^ with the FDC-marker CD35 inside B cell follicles (Figure 2C). Taken together, we found that tdTom^+^ TDA becomes closely associated with FDCs in TDLNs.

### Extensive scanning of tdTom^+^ FDCs by B cells

**Figure 3:**
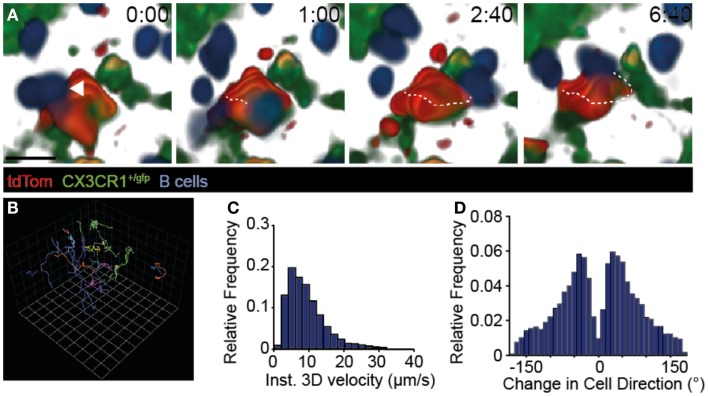
**Dynamic B cell behavior on TDA-coated FDCs**. **(A)** 2PM image sequence showing B cell migration along tdTom^+^ structures 31–95 μm below the TDLN capsule in CX3CR1^+/gfp^ recipients. A representative B cell track is shown as dotted line. Scale bar, 10 μm; time in minutes and seconds. **(B)** Representative tracks (>10 time points) of B cells migrating taken from the image sequence shown in A. One square corresponds to 11.2 μm. **(C)** Quantification of B cell speeds in TDLNs. **(D)** Quantification of turning angles of B cells in TDLNs. Data in **(A–D)** are from three mice in one experiment, with 433 B cell tracks analyzed.

Naïve B cells continuously migrate to B cell follicles, where they extensively scan the FDC network for presence of cognate Ag. In 2PM studies of TDLNs, we confirmed frequent interactions of adoptively transferred naïve B cells with tdTom^+^ FDCs (Figure 3A; Movies S3 and S4 in Supplementary Material). While some B cells were occasionally observed to arrest for a few minutes on such structures (see example in Figure 3A), most B cells showed robust motility as assessed by their track lengths (Figure 3B). Furthermore, B cells speeds (8.46 ± 2.52 μm/min; mean ± SD) (Figure 3C), turning angle distribution (Figure 3D), and their motility coefficient (16.7 μm^2^/min) in TDLNs were in the range of values obtained in non-tumor-draining resting LNs (32, 38–41). Taken together, our 2PM analysis uncovered extensive scanning of TDA by circulating naïve B cells, which is a prerequisite for the encounter of cognate Ag and the initiation of a humoral immune response.

### Whole-mount organ analysis uncovers widespread tdTom^+^ TDA deposition on FDCs of tumor-draining but not non-draining LNs

**Figure 4:**
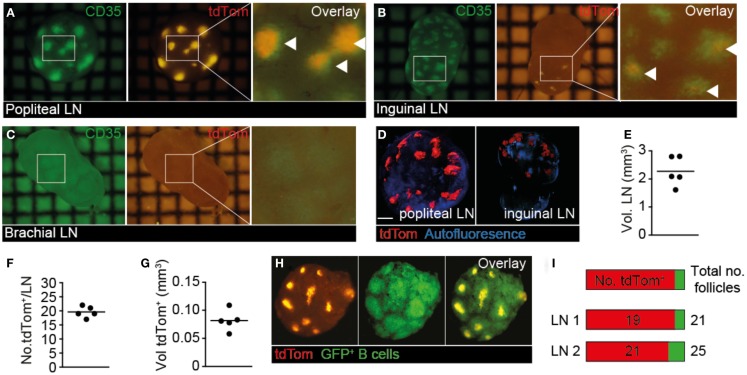
**Whole-mount analysis of tdTom^+^ spread in TDLN and non-TDLN**. B16.F10-tdTom^+^ cells were s.c. injected into C57BL/6 mice on day 0 and allowed to proliferate for 15 days. On day 14, Alexa488-labeled anti-CD35 mAb was injected s.c. into the same footpad to label FDCs. **(A)** Representative green (left) and red (right) fluorescent image of primary TDLN (popliteal LN). An overlay is shown in the right panel, with overlapping signal indicated by arrowheads. **(B)** Representative green (left) and red (right) fluorescent image of inguinal LN from the ipsilateral tumor side. An overlay is shown in the right panel, with overlapping signal indicated by arrowheads. **(C)** Representative green (left) and red (right) fluorescent image of non-draining brachial LN. An overlay is shown in the right panel. **(D)** Examples of 3D reconstructed OPT-derived autofluorescent and tdTom^+^ signals from popliteal and inguinal LNs. Scale bar, 400 μm. **(E)** Quantification of total TDLN volumes. Each dot represents one LN. **(F)** Number of tdTom^+^ structures per TDLN. **(G)** Quantification of total volume of tdTom^+^ structures per TDLN. **(H)** *Ex vivo* stereomicroscopic images of isolated TDLNs after transfer of GFP^+^ B cells 24 h before excision to identify B cell follicles. **(I)** Quantification of tdTom^+^ B cell follicles over total number of B cell follicles in two representative TDLNs. Each square in **(A–C)** represents 1 mm^2^. Data in **(A–C)** are representative of from 10 mice from 4 independent experiments, while data in **(D–I)** are representative for 3 mice from 3 independent experiments, with a total of 5 LNs analyzed.

In our live 2PM experiments, we observed a remarkably close association of TDA with FDCs. To gain a more comprehensive overview on TDA deposition in tumor-draining and non-draining LNs in a larger field of view than possible by 2PM, we isolated whole TDLN and non-draining LNs of mice, which had in addition received s.c. injections of the FDC-marker anti-CD35 mAb as tracer for fluorescent stereomicroscopic analysis (Figure 2B in Supplementary Material). In line with primary tumor growth in the footpad, we observed a strong tdTom^+^ signal in popliteal TDLNs, which colocalized with the anti-CD35^+^ signal (Figure 4A). In ipsilateral inguinal LNs, we observed a weaker tdTom^+^ signal co-localizing with CD35^+^ FDCs, which was present in some LNs in one hemisphere only, presumably because this hemisphere may have received lymph from other LNs downstream of TDLNs or directly from the footpad (42) (Figure 4B). In some experiments, we found tdTom^+^ signal in both hemispheres of ipsilateral inguinal LNs, but always in lower amounts than in the primary popliteal TDLN (not shown). In contrast, the non-draining brachial LN showed weak to no anti-CD35^+^ or tdTom^+^ signal (Figure 4C). Thus, our anti-CD35^+^ mAb tracer experiments mimic to a large extent the path followed by the surrogate tdTom TDA and suggest that the route of lymphatic drainage determines the location of TDA accumulation.

Since stereomicroscopic images showed qualitatively a widespread distribution of fluorescent TDA, we applied emission OPT to draining and non-draining LNs of mice, which had received s.c. B16.F10-tdTom injections in the right footpad, for a comprehensive assessment of tdTom^+^ signal frequency and distribution in entire organs (Figure 4D; Movie S5 in Supplementary Material) (28). When we analyzed the volume of TDLNs (Figure 4E), as well as the number and total volume of tdTom^+^ structures (Figures 4F,G), we found that tdTom^+^ structures occupied approximately 3.6% of the total TDLN volume distributed in approximately 20 locations per LN. Since our previous OPT analysis had determined that B cell follicles occupy approximately 10% of the total LN volume (28), and based on the observation that FDC occupy only the central part of B cell follicles, our data indicate that tdTom^+^ is deposited in most B cell follicles. To address this point directly, we adoptively transferred GFP^+^ B cells as markers for B cell follicles and repeated our OPT analysis (Figure 4H). As predicted, we observed a widespread tdTom^+^ signal distribution in the FDC-containing center of 84 and 90% of B cell follicles in two TDLNs (Figure 4I). Thus, at 4 weeks post s.c. B16.F10-tdTom injection, TDA occupied a large proportion of B cell follicles in TDLNs, providing an explanation for the high IgG titers found in serum 30 days after B16.F10-tdTom injection (Figure 1C). To the best of our knowledge, this analysis constitutes the first quantitative approach to assess antigen deposition on FDC, not only in cancer research but also more generally in the field of immunology.

### 2PM imaging uncovers that CX3CR1^+^ SCS macrophages colocalize with and take up subcellular-sized tdTom^+^ particles arriving in afferent lymph

**Figure 5:**
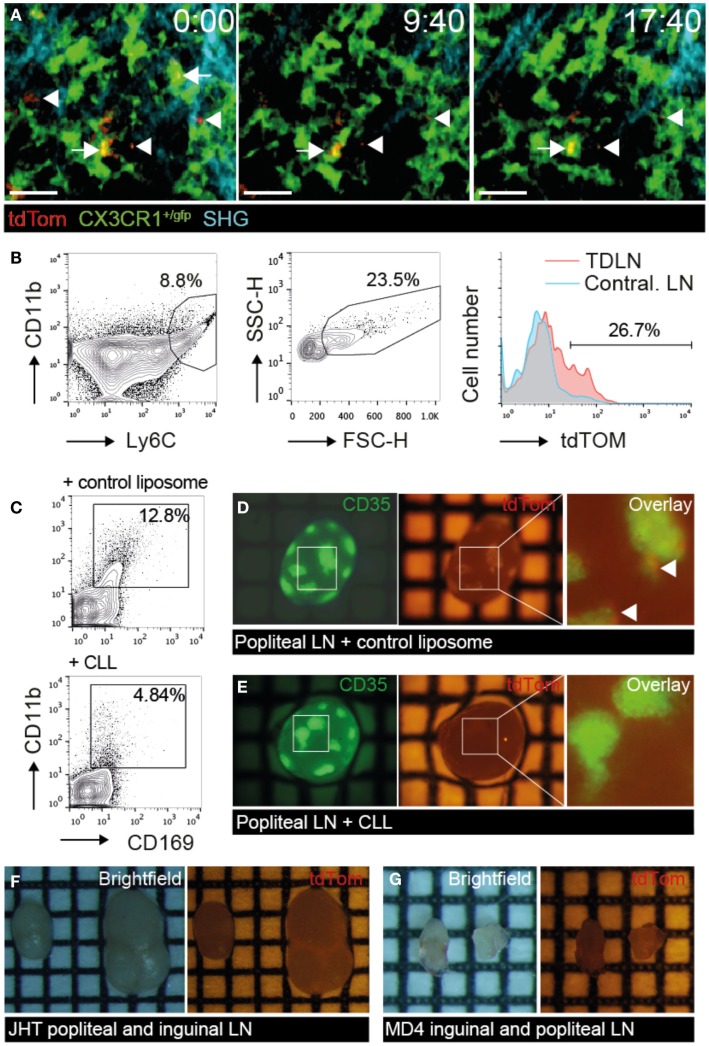
**tdTom^+^ particle capture by SCS macrophages correlates with TDA deposition on FDCs**. B16.F10-tdTom^+^ cells were s.c. injected into CX3CR1^+/gfp^, JHT or MD4 mice for 2PM and stereomicroscopy analysis. **(A)** 2PM image sequence of CX3CR1^+/gfp^ macrophages associating with subcellular-sized tdTom^+^ particles (arrows) in TDLN SCS on day 9 after tumor injection. Free tdTom^+^ particles are marked with an arrowhead. SHG, second harmonic generation image of collagen fibers in capsule. Scale bar, 20 μm; time in minutes and seconds. **(B)** Flow cytometry of CD11b^+^ Ly6C^+^ FCS^high^ macrophages for tdTom signal in TDLN and contralateral LN. **(C)** Representative flow cytometry analysis of popliteal TDLNs after control liposome or CLL treatment. **(D)** Representative green (left) and red (right) fluorescent image of primary TDLN (popliteal LN) in control liposome-treated mice on day 15 post tumor injection. Arrowheads in the overlay (right) point to tdTom^+^ signals. **(E)** Representative green (left) and red (right) fluorescent image of primary TDLN (popliteal LN) in CLL-treated mice. **(F)** Representative brightfield (left), and red fluorescent (right) image of popliteal and inguinal TDLN in B cell-deficient JHT mice. **(G)** Representative brightfield (left), and red fluorescent (right) image of inguinal and popliteal TDLN in MD4 mice. Each square in F represents 1 mm^2^. Data in **(A)** are representative of four mice in two independent experiments, while B is showing results of one of two mice. Data in **(C–G)** are representative of 3 CLL- and 2 control liposome-treated C57BL/6 mice in one experiment, and 7 JHT and 5 MD4 mice in 2 independent experiments on days 15 and 30 post tumor injection.

We next investigated the cellular mechanism of TDA capture in TDLN. Since SCS macrophages have been implicated in the uptake of apoptotic EG7 T cell thymoma tumor cells for presentation to T cells (23), we asked whether a similar process takes place in PLN draining s.c. injected B16.F10 melanoma cells. We surgically prepared TDLNs of CX3CR1^+/gfp^ mice, in which SCS macrophages are expressing GFP, for direct *in vivo* 2PM imaging 9–14 days post B16.F10-tdTom injection, when primary tumors became palpable in the footpad (Figure 5A; Figure S2A in Supplementary Material). We observed tdTom^+^ vesicles of subcellular size (<5 μm diameter) just below the collagen fibers of the LN capsule. These tdTom^+^ structures were typically in close apposition to or inside of CX3CR1^+^ macrophages in the SCS of the tumor-draining popliteal LNs (Figure 5A; Movie S6 in Supplementary Material). To verify that tdTom^+^ signal was indeed associated with macrophages, we used flow cytometry and identified a fraction of CD11b^+^ Ly6C^+^ macrophages that contained tdTom^+^ material in TDLNs but not in contralateral LNs (Figure 5B). Our data thus support a role for CX3CR1^+^ SCS macrophages in the uptake of subcellular-sized tumor-derived material presumably from apoptotic tumor cells, in line with previous observations (23).

### tdTom^+^ TDA deposition in TDLNs is abolished in the absence of macrophages, B cells or FDCs

Based on our observation of macrophage-mediated uptake of tdTom^+^ TDA, we used clodronate liposomes (CLL) or empty liposomes as controls to deplete macrophages systemically (Figure S2C in Supplementary Material). As predicted, CLL treatment abolished CD11b^high^ CD169^high^ macrophages in TDLNs to a large extent (Figure 5C). In parallel, we observed a strong decrease in tdTom^+^ TDA accumulation on FDCs of CLL but not control liposome-treated TDLNs (Figures 5D,E), supporting our intravital microscopy data that macrophages capture TDA arriving in afferent lymph. These data further suggest that other cell types such as migratory DCs cannot compensate for macrophages to deliver TDA to TDLNs.

Next, we investigated the efficacy of tdTom^+^ TDA deposition in TDLNs of JHT mice with defective B cell formation (43). In addition to lacking B cells, JHT mice do not develop FDCs, which allowed us to assess the function of these cells for TDA retention. Despite growth of B16.F10 tumor at the primary injection site in the footpad (not shown), we were unable to detect tdTom^+^ signal in TDLNs of JHT mice 15–30 days post tumor injection (Figure 5F). Similar results were obtained in MD4 BCR x RAG^−/−^ transgenic mice, in which B cells are restricted to a single specificity of antibodies. While the lack of T cells resulted in smaller LN size, no tdTom^+^ signal was detected despite normal primary tumor growth in footpad (Figure 5G). Taken together, the lack of tdTom^+^ signals in macrophage-depleted or B cell- and FDC-deficient TDLNs suggests that the majority of tdTom^+^ signal is indeed taken up by macrophages and becomes associated with FDCs, although we cannot exclude occasional metastatic B16.F10-tdTom cells contributing to red fluorescent signal in TDLNs.

## Discussion

Lymph nodes filter afferent lymph for the presence of noxious particles to prevent their systemic spread and for presentation of antigen to passing lymphocytes. In the present study, we combined 2PM and OPT imaging to dissect the mechanism by which unprocessed, or incompletely processed, TDA accumulates in TDLNs. To this end, we took advantage of an intrinsically fluorescent surrogate TDA as a tool to examine its spread in secondary lymphoid organs, following previously established protocols investigating fluorescently labeled virus particle uptake (16). We report two major findings: first, TDA is efficiently captured by TDLNs and to a minor extent by further downstream LNs for an unexpectedly widespread distribution on most FDC networks. Second, we were able to correlate this finding with intravital imaging of SCS macrophage association with subcellular-sized TDA^+^ particles. Macrophage depletion abolished TDA uptake in TDLNs, providing strong support that SCS macrophages, rather than migratory DCs or passive diffusion, are required for efficient capture of tumor-derived particles in our model.

While confocal and multiphoton microscopy have become central tools for studying metastazing cancer cells and interactions with infiltrating lymphocytes (44–47), the present study represents to the best of our knowledge the first attempt to exploit fluorescent imaging to comprehensively examine TDA deposition in draining LNs. While in our mouse model, we at first suspected that tdTom^+^ signals in TDLNs stemmed directly from metastasizing B16.F10-tdTom melanoma cells, as described in models of i.v. injected GFP-expressing melanoma cells (48), several lines of evidence suggested that this signal derived from TDA: first, we observed macrophage capture of presumably apoptotic subcellular tdTom^+^ fragments, with subsequent accumulation on FDC. Second, at later time points, parenchymal tdTom^+^ signals overlapped with the FDC-marker CD35 and assumed the same dendritic shape as FDC by immunofluorescence and 2PM analysis. Third, FDC, B cells, and/or macrophages were required for tdTom^+^ signal accumulation inside TDLNs. Finally, we were unable to recover tumor cells from TDLNs, in contrast to the primary tumor site (not shown). Taken together, we postulate that tdTom^+^ signals observed on FDCs represent bona fide TDA and support the use of B16.F10-tdTom cells as novel tool to quantitatively follow TDA deposition in TDLNs.

Our combined 2PM and OPT analysis uncovered two relevant aspects of TDA presentation, which have gone previously unnoticed: first, we show extensive scanning of FDC-deposited TDA by circulating B cells. In our experimental setup, this scanning did not induce significant slowing of B cells as is the case when cognate Ag is encountered in inflamed LNs (20, 21). This is most likely because the Ag-specific precursor frequency in our polyclonal B cell population was too low to identify occasional responders. A second central aspect is the OPT-based evaluation of TDA spread, which showed that approximately 90% of FDC networks within B cell follicles contained abundant TDA. This spread was not limited to the first TDLN but spilled over to further LNs. In combination with our observation of extensive B cell scanning of TDA-bearing FDCs, it is likely that a sizeable percentage, if not the vast majority, of the naïve B cell pool comes in direct contact with TDA during the first weeks after tumor injection. This may explain the readily detectable anti-TDA IgG levels in tumor-bearing mice.

Using 2PM imaging, we found that CX3CR1^+^ SCS macrophages take up subcellular-sized particles containing a cytoplasmic fluorescent fusion protein produced by B16 melanoma cells, which may represent apoptotic bodies from the primary tumor site or tumor-derived microvesicles (49). A similar mechanism of apoptotic vesicle uptake has been described to involve CD169^+^ macrophages with cross-presenting capacity for CD8^+^ T cell activation (23). Yet, the preservation of tdTom signal over weeks suggests that not all engulfed TDA is cross-presented in our model, but deposited and/or endocytosed as largely unprocessed antigen on FDCs (37). It has to be kept in mind that our study does not address whether TDA travels freely from primary tumors via afferent lymph or is transported by CX3CR1^−^ DCs or macrophage subsets. In any case, our data extend the functions of SCS macrophages to not only induce T cell responses but also to capture Ag for presentation to B cells. In fact, this process has been shown to occur frequently during viral infections, and has been associated with non-specific B cell capture of immune complexes as shuttle from macrophages to FDCs (25). Along this line, we observed a lack of TDA capture in MD4 LNs, suggesting a role for immune complexes for TDA relay to FDCs. In a recent study, SCS macrophage depletion resulted in blunted B cell responses in draining LNs, owing to defective antigen capture (50). While we have not specifically addressed this issue in our study, it is likely that CLL treatment prevents B cell activation in TDLNs in a similar fashion. Under these conditions, TDA may then be further drained by lymphatic vessels, until it becomes deposited by mechanisms independent of macrophage capture, e.g., by DC subsets. Thus, CLL treatment may simply alter the site of antibody production, rather than suppress humoral responses, as has been shown in a viral infection model (24). Future experiments using adoptively transferred BCR-transgenic B cells and appropriate tumor antigens need to be performed to shed light on this important aspect.

In sum, our microscopic and whole-mount data uncover a previously unnoticed widespread macrophage-mediated TDA deposition on FDC in the B16.F10 model, using an intrinsically fluorescent fusion protein as surrogate TDA. Extensive TDA scanning by naïve B cells correlated with anti-TDA IgG production. More than 20 years ago, TDA deposition on FDC has been reported in TDLNs of colon carcinoma patients (51), which we confirm here in TLDNs of melanoma patients. In future studies, we will expand the panel of tumor antigens to generate a comprehensive map of the TDA, which are most frequently associated with B cell follicles in melanoma patients. Such data may be useful in future anticancer immunotherapies, in particular since recent patient studies have shown that the abundance of immune cells governing humoral responses positively correlates with good prognosis (10, 11).

## Author Contributions

FM and JS performed and analyzed mouse experiments; CS analyzed human samples; SP, RS, and MD provided vital material. FM and JS wrote the manuscript with important input by all coauthors.

## Conflict of Interest Statement

The authors declare that the research was conducted in the absence of any commercial or financial relationships that could be construed as a potential conflict of interest.

## Supplementary Material

The Supplementary Material for this article can be found online at http://www.frontiersin.org/Journal/10.3389/fimmu.2015.00114/abstract

Figure S1 **MelA deposition in B cell follicles of TDLNs taken from human melanoma patients**. **(A–C)** Immunofluorescent analysis of CD20, MelA, and DAPI signals in TDLNs from human melanoma patients. In **(B,C)**, representative MelA^+^ positive signals associated with B cell follicles are marked by arrows, while in **(A)**, arrows point to MelA^−^ B cell follicles. Scale bar, 100 μm. Sections are representative of five sections from three donors.

Figure S2 **Experimental schemes for 2PM and whole-mount LN imaging**. **(A)** Scheme for 2PM experiments. **(B)** Scheme for 3D whole-mount quantification of TDA deposition. **(C)** Scheme for macrophage depletion experiments using control and clodronate liposomes.

Movie S1 **2PM image sequence of tdTom^+^ structures below SCS of TDLNs**. tdTom^+^ structures (red) in close proximity to SCS macrophages (green-blue). Co-localization of tdTom^+^ and LYVE-1^+^ signal (green) appears yellow. Time in minutes and seconds.

Movie S2 **High-resolution image sequence of dendritic tdTom+ structure in TDLNs**. tdTom^+^ structures (red; arrows) in close proximity to macrophages (green-blue) show dendritic morphology. Scale bar, 20 μm; time in minutes and seconds.

Movie S3 **tdTom^+^ structure scanning by naïve B cells**. tdTom^+^ structures (red) in close proximity to macrophages (green) are scanned by naïve B cells (blue) within TDLNs. Representative B cell tracks are shown in white, while arrows show immobile macrophages for comparison. Scale bar, 30 μm; time in minutes and seconds.

Movie S4 **tdTom^+^ structure scanning by naïve B cells**. tdTom^+^ structures (red) in close proximity to macrophages (green) are scanned by naïve B cells (blue) within TDLNs. To highlight interactions, the image sequence is kept in 3D mode and zooms into a tdTom^+^-rich region. Time in minutes and seconds.

Movie S5 **OPT reconstruction of tdTom^+^ structure in TDLNs**. tdTom^+^ structures (red) are concentrated around the periphery of TDLN (autofluorescent signal in blue). Scale bar, 400 μm.

Movie S6 **tdTom^+^ fragment retention in SCS of TDLNs**. tdTom^+^ particles (red) localize inside (arrow) or in close proximity (arrowhead) of CX3CR1^+^ SCS macrophages (green) located below the collagen fibers (blue) of the TDLN capsule. Scale bar, 20 μm; time in minutes and seconds.

## References

[1] Lewis MG Ikonopisov RL Nairn RC Phillips TM Fairley GH Bodenham DC Tumour-specific antibodies in human malignant melanoma and their relationship to the extent of the disease. Br Med J (1969) 3:547–52. 10.1136/bmj.3.5670.547 4896110 PMC1984348

[2] Gilbert AE Karagiannis P Dodev T Koers A Lacy K Josephs DH Monitoring the systemic human memory B cell compartment of melanoma patients for anti-tumor IgG antibodies. PLoS One (2011) 6:e19330. 10.1371/journal.pone.0019330 21559411 PMC3084832

[3] Zhang Y Morgan R Podack ER Rosenblatt J. B cell regulation of anti-tumor immune response. Immunol Res (2013) 57:115–24. 10.1007/s12026-013-8472-1 24293009

[4] Li Q Lao X Pan Q Ning N Yet J Xu Y Adoptive transfer of tumor reactive B cells confers host T-cell immunity and tumor regression. Clin Cancer Res (2011) 17:4987–95. 10.1158/1078-0432.CCR-11-0207 21690573 PMC3149727

[5] Couzin-Frankel J Cancer immunotherapy. Science (2013) 342:1432–3 10.1126/science.342.6165.1432 24357284

[6] Vesely MD Kershaw MH Schreiber RD Smyth MJ. Natural innate and adaptive immunity to cancer. Annu Rev Immunol (2011) 29:235–71. 10.1146/annurev-immunol-031210-101324 21219185

[7] Chen DS Mellman I. Oncology meets immunology: the cancer-immunity cycle. Immunity (2013) 39:1–10. 10.1016/j.immuni.2013.07.012 23890059

[8] Galon J Costes A Sanchez-Cabo F Kirilovsky A Mlecnik B Lagorce-Pagès C Type, density, and location of immune cells within human colorectal tumors predict clinical outcome. Science (2006) 313:1960–4. 10.1126/science.1129139 17008531

[9] Galon J Angell HK Bedognetti D Marincola FM. The continuum of cancer immunosurveillance: prognostic, predictive, and mechanistic signatures. Immunity (2013) 39:11–26. 10.1016/j.immuni.2013.07.008 23890060

[10] Bindea G Mlecnik B Tosolini M Kirilovsky A Waldner M Obenauf AC Spatiotemporal dynamics of intratumoral immune cells reveal the immune landscape in human cancer. Immunity (2013) 39:782–95. 10.1016/j.immuni.2013.10.003 24138885

[11] Gu-Trantien C Loi S Garaud S Equeter C Libin M de Wind A CD4^+^ follicular helper T cell infiltration predicts breast cancer survival. J Clin Invest (2013) 123:2873–92. 10.1172/JCI67428 23778140 PMC3696556

[12] Ali HR Provenzano E Dawson SJ Blows FM Liu B Shah M Association between CD8+ T-cell infiltration and breast cancer survival in 12 439 patients. Ann Oncol (2014) 25(8):1536–43. 10.1093/annonc/mdu191 24915873

[13] Tumeh PC Harview CL Yearley JH Shintaku IP Taylor EJM Robert L PD-1 blockade induces responses by inhibiting adaptive immune resistance. Nature (2014) 515:568–71. 10.1038/nature13954 25428505 PMC4246418

[14] León B Ballesteros-Tato A Randall TD Lund FE. Prolonged antigen presentation by immune complex-binding dendritic cells programs the proliferative capacity of memory CD8 T cells. J Exp Med (2014) 211:1637–55. 10.1084/jem.20131692 25002751 PMC4113940

[15] Guo K Li J Tang JP Tan CPB Hong CW Al-Aidaroos AQO Targeting intracellular oncoproteins with antibody therapy or vaccination. Sci Transl Med (2011) 3:99ra85. 10.1126/scitranslmed.3002296 21900592

[16] Gonzalez SF Degn SE Pitcher LA Woodruff M Heesters BA Carroll MC. Trafficking of B cell antigen in lymph nodes. Annu Rev Immunol (2011) 29:215–33. 10.1146/annurev-immunol-031210-101255 21219172

[17] Qi H Egen JG Huang AYC Germain RN. Extrafollicular activation of lymph node B cells by antigen-bearing dendritic cells. Science (2006) 312:1672–6. 10.1126/science.1125703 16778060

[18] Roozendaal R Mempel TR Pitcher LA Gonzalez SF Verschoor A Mebius RE Conduits mediate transport of low-molecular-weight antigen to lymph node follicles. Immunity (2009) 30:264–76. 10.1016/j.immuni.2008.12.014 19185517 PMC2699624

[19] Heesters BA Myers RC Carroll MC Follicular dendritic cells: dynamic antigen libraries. Nat Rev Immunol (2014) 14:495–504 10.1038/nri3689 24948364

[20] Junt T Moseman EA Iannacone M Massberg S Lang PA Boes M Subcapsular sinus macrophages in lymph nodes clear lymph-borne viruses and present them to antiviral B cells. Nature (2007) 450:110–4. 10.1038/nature06287 17934446

[21] Carrasco YR Batista FD. B cells acquire particulate antigen in a macrophage-rich area at the boundary between the follicle and the subcapsular sinus of the lymph node. Immunity (2007) 27:160–71. 10.1016/j.immuni.2007.06.007 17658276

[22] Gray EE Cyster JG. Lymph node macrophages. J Innate Immun (2012) 4:424–36. 10.1159/000337007 22488251 PMC3574571

[23] Asano K Nabeyama A Miyake Y Qiu C-H Kurita A Tomura M CD169-positive macrophages dominate antitumor immunity by crosspresenting dead cell-associated antigens. Immunity (2011) 34:85–95. 10.1016/j.immuni.2010.12.011 21194983

[24] Gonzalez SF Lukacs-Kornek V Kuligowski MP Pitcher LA Degn SE Kim Y-A Capture of influenza by medullary dendritic cells via SIGN-R1 is essential for humoral immunity in draining lymph nodes. Nat Immunol (2010) 11:427–34. 10.1038/ni.1856 20305659 PMC3424101

[25] Phan TG Green JA Gray EE Xu Y Cyster JG. Immune complex relay by subcapsular sinus macrophages and noncognate B cells drives antibody affinity maturation. Nat Immunol (2009) 10:786–93. 10.1038/ni.1745 19503106 PMC2776777

[26] Baniyash M Smorodinsky NI Yaakubovicz M Witz IP. Serologically detectable MHC and tumor-associated antigens on B16 melanoma variants and humoral immunity in mice bearing these tumors. J Immunol (1982) 129:1318–23. 7108209

[27] Proulx ST Luciani P Christiansen A Karaman S Blum KS Rinderknecht M Use of a PEG-conjugated bright near-infrared dye for functional imaging of rerouting of tumor lymphatic drainage after sentinel lymph node metastasis. Biomaterials (2013) 34:5128–37. 10.1016/j.biomaterials.2013.03.034 23566803 PMC3646951

[28] Kumar V Scandella E Danuser R Onder L Nitschké M Fukui Y Global lymphoid tissue remodeling during a viral infection is orchestrated by a B cell-lymphotoxin-dependent pathway. Blood (2010) 115:4725–33. 10.1182/blood-2009-10-250118 20185585

[29] Gu H Zou Y-R Rajewsky K Independent control of immunoglobulin switch recombination at individual switch regions evidenced through Cre-loxP-mediated gene targeting. Cell (1993) 73:1155–64 10.1016/0092-8674(93)90644-6 8513499

[30] Goodnow CC Crosbie J Adelstein S Lavoie TB Smith-Gill SJ Brink RA Altered immunoglobulin expression and functional silencing of self-reactive B lymphocytes in transgenic mice. Nature (1988) 334:676–82. 10.1038/334676a0 3261841

[31] Jung S Aliberti J Graemmel P Sunshine MJ Kreutzberg GW Sher A Analysis of fractalkine receptor CX(3)CR1 function by targeted deletion and green fluorescent protein reporter gene insertion. Mol Cell Biol (2000) 20:4106–14. 10.1128/MCB.20.11.4106-4114.2000 10805752 PMC85780

[32] Coelho FM Natale D Soriano SF Hons M Swoger J Mayer J Naive B-cell trafficking is shaped by local chemokine availability and LFA-1-independent stromal interactions. Blood (2013) 121:4101–9. 10.1182/blood-2012-10-465336 23558016

[33] Nombela-Arrieta C Mempel TR Soriano SF Mazo I Wymann MP Hirsch E A central role for DOCK2 during interstitial lymphocyte motility and sphingosine-1-phosphate-mediated egress. J Exp Med (2007) 204:497–510. 10.1084/jem.20061780 17325199 PMC2137902

[34] Mempel TR Henrickson SE Andrian Von UH. T-cell priming by dendritic cells in lymph nodes occurs in three distinct phases. Nature (2004) 427:154–9. 10.1038/nature02238 14712275

[35] Hama H Kurokawa H Kawano H Ando R Shimogori T Noda H Scale: a chemical approach for fluorescence imaging and reconstruction of transparent mouse brain. Nat Neurosci (2011) 14(11):1481–8. 10.1038/nn.2928 21878933

[36] Schlapbach C Zawodniak A Irla N Adam J Hunger RE Yerly D NKp46+ cells express granulysin in multiple cutaneous adverse drug reactions. Allergy (2011) 66:1469–76. 10.1111/j.1398-9995.2011.02677.x 21819408

[37] Heesters BA Chatterjee P Kim Y-A Gonzalez SF Kuligowski MP Kirchhausen T Endocytosis and recycling of immune complexes by follicular dendritic cells enhances B cell antigen binding and activation. Immunity (2013) 38:1164–75. 10.1016/j.immuni.2013.02.023 23770227 PMC3773956

[38] Miller MJ Wei SH Parker I Cahalan MD. Two-photon imaging of lymphocyte motility and antigen response in intact lymph node. Science (2002) 296:1869–73. 10.1126/science.1070051 12016203

[39] Han S-B Moratz C Huang N-N Kelsall B Cho H Shi C-S Rgs1 and Gnai2 regulate the entrance of B lymphocytes into lymph nodes and B cell motility within lymph node follicles. Immunity (2005) 22:343–54. 10.1016/j.immuni.2005.01.017 15780991

[40] Boscacci RT Pfeiffer F Gollmer K Sevilla AIC Martin AM Soriano SF Comprehensive analysis of lymph node stroma-expressed Ig superfamily members reveals redundant and nonredundant roles for ICAM-1, ICAM-2, and VCAM-1 in lymphocyte homing. Blood (2010) 116:915–25. 10.1182/blood-2009-11-254334 20395417 PMC3324225

[41] Park C Hwang I-Y Sinha RK Kamenyeva O Davis MD Kehrl JH. Lymph node B lymphocyte trafficking is constrained by anatomy and highly dependent upon chemoattractant desensitization. Blood (2012) 119:978–89. 10.1182/blood-2011-06-364273 22039261 PMC3271721

[42] Harrell MI Iritani BM Ruddell A. Lymph node mapping in the mouse. J Immunol Methods (2008) 332:170–4. 10.1016/j.jim.2007.11.012 18164026 PMC2342937

[43] Fu YX Huang G Wang Y Chaplin DD. B lymphocytes induce the formation of follicular dendritic cell clusters in a lymphotoxin alpha-dependent fashion. J Exp Med (1998) 187:1009–18. 10.1084/jem.187.7.1009 9529317 PMC2212211

[44] Engelhardt JJ Boldajipour B Beemiller P Pandurangi P Sorensen C Werb Z Marginating dendritic cells of the tumor microenvironment cross-present tumor antigens and stably engage tumor-specific T cells. Cancer Cell (2012) 21:402–17. 10.1016/j.ccr.2012.01.008 22439936 PMC3311997

[45] Condeelis J Segall JE Intravital imaging of cell movement in tumours. Nat Rev Cancer (2003) 3:921–30 10.1038/nrc1231 14737122

[46] Beerling E Ritsma L Vrisekoop N Derksen PWB van Rheenen J. Intravital microscopy: new insights into metastasis of tumors. J Cell Sci (2011) 124:299–310. 10.1242/jcs.072728 21242309 PMC3021994

[47] Kienast Y Baumgarten von L Fuhrmann M Klinkert WEF Goldbrunner R Herms J Real-time imaging reveals the single steps of brain metastasis formation. Nat Med (2009) 16:116–22. 10.1038/nm.2072 20023634

[48] Yang M Jiang P An Z Baranov E Li L Hasegawa S Genetically fluorescent melanoma bone and organ metastasis models. Clin Cancer Res (1999) 5:3549–59. 10589771

[49] Pucci F Pittet MJ. Molecular pathways: tumor-derived microvesicles and their interactions with immune cells in vivo. Clin Cancer Res (2013) 19:2598–604. 10.1158/1078-0432.CCR-12-0962 23426276 PMC3655093

[50] Gaya M Castello A Montaner B Rogers N Reis e Sousa C Bruckbauer A Inflammation-induced disruption of SCS macrophages impairs B cell responses to secondary infection. Science (2015) 347:667–72. 10.1126/science.aaa1300 25657250

[51] Mariani-Costantini R Muraro R Ficari F Valli C Bei R Tonelli F Immunohistochemical evidence of immune responses to tumor-associated antigens in lymph nodes of colon carcinoma patients. Cancer (1991) 67:2880–6. 10.1002/1097-0142(19910601)67:11<2880::AID-CNCR2820671129>3.0.CO;2-A 1709062

