# Supplementary material for: Intravital and Whole-Organ Imaging Reveals Capture of Melanoma-Derived Antigen by Lymph Node Subcapsular Macrophages Leading to Widespread Deposition on Follicular Dendritic Cells
Source: Front Immunol. 2015 Mar 13;6:114. doi: 10.3389/fimmu.2015.00114 (PMC4358226; doi:10.3389/fimmu.2015.00114)
Supplement: Figure S1 — MelA deposition in B cell follicles of TDLNs taken from human melanoma patients. (A–C) Immunofluorescent analysis of CD20, MelA, and DAPI signals in TDLNs from human melanoma patients. In (B,C), representative MelA+ positive signals associated with B cell follicles are marked by arrows, while in (A), arrows point to MelA− B cell follicles. Scale bar, 100 μm. Sections are representative of five sections from three donors. [file Image_1.PDF]

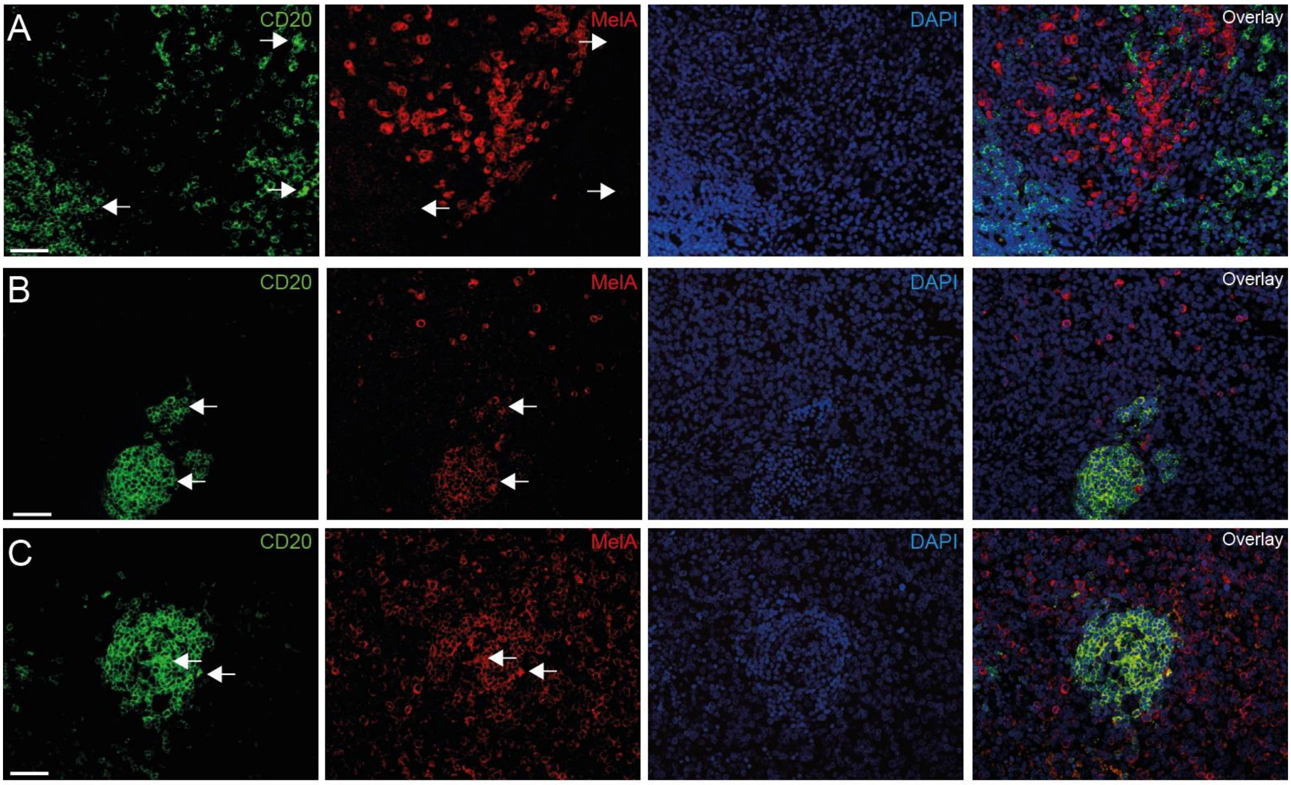

Supplemental Figure 1

MelA deposition in B cell follicles of TDLNs taken from human melanoma patients. A - C. Immunofluorescent analysis of CD20, MelA and DAPI signals in TDLNs from human melanoma patients. In B and C, representative MelA$^+$ positive signals associated with B cell follicles are marked by arrows, while in A, arrows point to MelA$^-$ B cell follicles. Scale bar, 100 µm. Sections are representative of 5 sections from 3 donors.
