# Supplementary material for: Intravital and Whole-Organ Imaging Reveals Capture of Melanoma-Derived Antigen by Lymph Node Subcapsular Macrophages Leading to Widespread Deposition on Follicular Dendritic Cells
Source: Front Immunol. 2015 Mar 13;6:114. doi: 10.3389/fimmu.2015.00114 (PMC4358226; doi:10.3389/fimmu.2015.00114)
Supplement: Figure S2 — Experimental schemes for 2PM and whole-mount LN imaging. (A) Scheme for 2PM experiments. (B) Scheme for 3D whole-mount quantification of TDA deposition. (C) Scheme for macrophage depletion experiments using control and clodronate liposomes. [file Image_2.PDF]

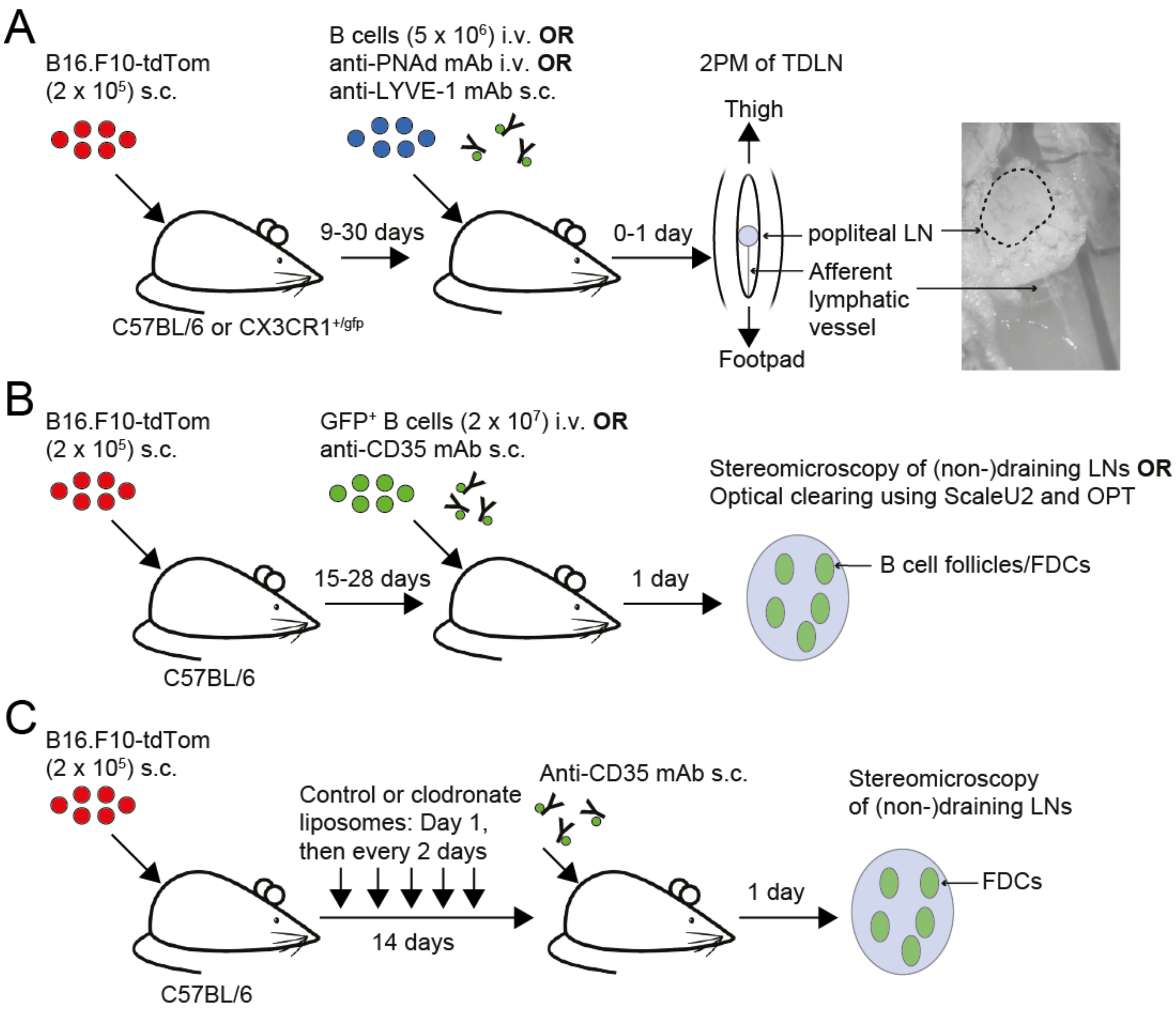

Supplemental Figure 2

Experimental schemes for 2PM and whole mount LN imaging. A. Scheme for 2PM experiments. **B.** Scheme for 3D whole-mount quantification of TDA deposition. **C.** Scheme for macrophage depletion experiments using control and clodronate liposomes.
